# Editorial: Microbial Response to a Rapidly Changing Marine Environment: Global Warming and Ocean Acidification

**DOI:** 10.3389/fmicb.2021.731732

**Published:** 2021-08-09

**Authors:** Mi Sun Yun, Jun Sun, Connie Lovejoy, Sang Heon Lee

**Affiliations:** ^1^College of Marine and Environmental Sciences, Tianjin University of Science and Technology, Tianjin, China; ^2^College of Marine Science and Technology, China University of Geosciences Wuhan, Wuhan, China; ^3^Département de Biologie, Université Laval, Quebec, QC, Canada; ^4^Department of Oceanography, Pusan National University, Busan, South Korea

**Keywords:** microbial community, marine environment, ecosystem, warming, ocean acidification

The World Ocean is undergoing rapid and substantial changes, particularly, in terms of warming and acidification. Global sea surface temperatures have risen by 0.7°C in recent years (2005–2014) relative to pre-industrial times (1870–1899) (Gattuso et al., 2015). The ocean pH is decreasing in response to the increasing anthropogenic CO_2_ emissions (Ocean Acidification; OA). This clear trend of ocean warming and acidification was documented in the fifth Assessment Report (AR5) by Intergovernmental Panel on Climate Change (IPCC). The warming of the surface ocean could increase stratification, suppress transport of nutrients into the upper photic zone and alter hydrographic properties or patterns of ocean circulation. The effects of OA include the changes of seawater carbonate chemistry such as an increase in partial pressure of seawater CO_2_ (*p*CO_2_) concentration and a decrease in calcium carbonate (CaCO_3_) saturation state (Royal Society, 2005; IPCC, 2014). Consequently, the warming and acidification will have substantial impacts on the growth and survival of marine organisms (e.g., Brander, 2010; Doney et al., 2012; Hollowed et al., 2013). In particular, microbes, as a vital component of the marine ecosystem that includes microalga (phytoplankton), protists, fungi, viruses, and the two main groups of prokaryotes (Bacteria and Archaea), are vulnerable to these environmental changes. Since these microscopic organisms drive major biogeochemical cycles and support higher food webs globally, physiological, and ecological alterations in microbial communities caused by marine environmental changes can herald changes not only in pathways of energy transfer through the food web but also in global biogeochemical cycles. Considering the microbial communities' pivotal roles in the marine ecosystem and biogeochemical cycles, it is important to understand recent changes in microbial communities and how future changes might arise under the ongoing environmental forcing of the warming and acidifying oceans.

The goal of this Research Topic was to collect studies on present and future possible changes in microbial communities with respect to environmental change and their consequences within various oceans. The topic contains 10 diverse scientific contributions on many fundamental questions related to microbial communities in oceanic environments, and report on physiological and ecological responses of microbial communities to environmental changes. The studies from a range of geographic regions were collected, from more coastal systems to open oceans, and include Tera Nova Bay in Antarctica, Mediterranean waters, the East/Japan Sea, the South China Sea, the Western Pacific Ocean, the South Pacific Ocean, and the Ross Sea of the Southern Ocean. In the context of taxonomic diversity, heterotrophic bacteria, viruses, microalga, and other prokaryotes were included in this Research Topic.

Out of the 10 works published, seven of them are focused on the phytoplankton communities, or single species of microalgae, or environmental drivers. Kim et al. describe the seasonal variability in the macromolecular composition of the sea surface in front of the Korean station in Terra Nova Bay, Ross Sea, Antarctica. They show changes in organic matter composition mainly derived by changes in phytoplankton metabolism between the productive and the non-productive season. Their study helps us to understand the effect of future climate change on the composition of organic matter derived from phytoplankton in the polar oceans. Biochemical compositions such as macromolecular and amino acids of phytoplankton in the Ross Sea, Antarctica was investigated by Jo et al.. They found distinct differences in the biochemical composition between two major bloom-forming phytoplankton groups (diatoms and *Phaeocystis antarctica*). They discuss how these different compositions may highlight the different strategies of these two phytoplankton communities to cope with ongoing environmental changes in the Antarctic Ocean. Kang et al. demonstrate the differences in carbon uptake rates and intracellular biochemical compositions between two different size fractions of phytoplankton to understand the ecological roles of the small phytoplankton in terms of food quantity and quality in the East/Japan Sea where the water temperature has rapidly increased. Their findings show that the increase of small phytoplankton under the warming ocean conditions could negatively affect the primary productivity and caloric content, with further consequences on the marine food webs. The photosynthetic responses to oceanic physio-chemical conditions and phytoplankton communities in the oligotrophic Western Pacific Ocean were presented by Wei et al.. Their study found that the important biotic variables influencing Fv/Fm are diatoms, Prochlorococcus, and picoeukaryotes, whilst the maximum of primary production is closely related to cyanobacteria, dinoflagellates, and *Synechococcus*. The detailed investigation of a chromophytic phytoplankton community using high-throughput sequencing of rbcL genes in the Western Pacific Ocean was presented by Pujari et al.. The authors found that the diversity of RuBisCO encoding rbcL gene varies with depth and across latitudes in the Western Pacific Ocean. The variation observed in chromophytic phytoplankton suggests the strong influence of environmental variables on biological production induced by oceanographic features. Thangaraj et al. investigated comprehensive proteomic profiling of diatom *Skeletonema dohrnii* with a change of temperature and silicate deprivation based on the iTRAQ proteomic approach, to understand the effect of the temperature and nutrient on the physiology of marine diatoms growth and photosynthesis. Their study shows that the proteome analysis for environmental stress-response of diatoms could extend our understanding for the potential impacts of climate change on the physiological adjustment to the metabolic process of phytoplankton. Sow et al. present a clear and concise description of the biogeography of *Phaeocystis* along a transect from the ice edge to the equator in the South Pacific Ocean, by way of high-throughput 18S rRNA gene sequencing. Their study shows that *Phaeocystis* could be occasionally highly abundant and diverse in the South Pacific Ocean, whereas the oceanic fronts could be the driving force for the distribution and structure of *Phaeocystis* assemblages in the ocean. Their work greatly expands our knowledge about the biodiversity patterns and abundances of *Phaeocystis* as a globally important nano-eukaryote.

Three of the 10 published works have focused on the response of viruses, prokaryotes or microbial communities to environmental stress. The response of viruses to two anthropogenic stressors (OA and eutrophication) was presented by Malits et al.. Their study demonstrates that the effect of OA on viral dynamics and viral-mediated mortality varies depending on the nutrient regime of the studied systems. It helps us to understand how viral-mediated mortality of microbes (VMMM) can be modified with environmental forcing. Another work looks into the contribution of grazers and viruses in controlling ecologically distinct prokaryotic sub-groups (i.e., high nucleic acid (HNA) and low nucleic acid (LNA) cells) along a cross-shore nutrient gradient in the northern South China Sea (Hu et al.). Their study shows how the nutrient regime influences the fate of ecologically relevant prokaryotic groups in the actual context of global warming and the anticipated oligotrophication of the future ocean. Sörenson et al. address the resilience of a marine microbial community, cultivated in an outdoor photobioreactor, when exposed to a naturally occurring seasonal stress. Differential gene expression analyses suggest that community function at warm temperatures is based on concomitant utilization of inorganic and organic carbon assigned to autotrophs and heterotrophs, while at colder temperatures, the uptake of organic carbon was performed primarily by autotrophs. Overall, the microbial community maintains a similar level of diversity and function within and across autotrophic and heterotrophic levels, confirming the cross-scale resilience theory.

The topics of the papers published in this Research Topic range from viruses, prokaryotes to phytoplankton and cover microbial communities from the various oceans. The studies confirm that the changes already occurring in ocean environments affect the metabolism and physiology of microbial communities, and further suggest that future changes will impact the physiological and ecological function or strategy of the microbial community in the marine ecosystem. Since most of these studies focus on the response of a single taxonomic population of microbes to environmental changes, our special issue highlights the need for studies to understand how ecological interactions occurring within and among the microbial community in the changing ocean will affect ecosystem structure and function. Finally, we hope that the group of papers that we have drawn together here will be a valuable addition to the accumulating observational evidence of how microbial communities are responding to the climate-related changes and consequently useful for evaluating and predicting the ongoing and future responses of marine ecosystems associated with the global climate change.

## Author Contributions

MSY wrote the text with input from CL. All other authors commented on and approved the text.

## Conflict of Interest

The authors declare that the research was conducted in the absence of any commercial or financial relationships that could be construed as a potential conflict of interest.

## Publisher's Note

All claims expressed in this article are solely those of the authors and do not necessarily represent those of their affiliated organizations, or those of the publisher, the editors and the reviewers. Any product that may be evaluated in this article, or claim that may be made by its manufacturer, is not guaranteed or endorsed by the publisher.
